# Crystal structure of 5,7,12,14-tetra­hydro-5,14:7,12-bis­([1,2]benzeno)­penta­cene-6,13-dione^1^

**DOI:** 10.1107/S2056989016017461

**Published:** 2016-11-04

**Authors:** Mohammad Nozari, Jerry P. Jasinski, Manpreet Kaur, Anthony W. Addison, Ahmad Arabi Shamsabadi, Masoud Soroush

**Affiliations:** a Department of Chemistry, Drexel University, 3141 Chestnut St., Philadelphia, PA 19104, USA; b Department of Chemistry, Keene State College, 229 Main Street, Keene, NH 03435, USA; c Department of Chemical and Biological Engineering, Drexel University, 3141 Chestnut St., Philadelphia, PA 19104, USA

**Keywords:** crystal structure, iptycene, pentiptycene, polymers of intrinsic microporosity (PIM), quinone, voltammetry

## Abstract

The lattice of 5,7,12,14-tetra­hydro-5,14:7,12-bis­([1,2]benzeno)­penta­cene-6,13-dione, C_34_H_20_O_2_, at 173 K has triclinic (*P*-1) symmetry and crystallizes with four independent half-mol­ecules in the asymmetric unit. Each mol­ecule is generated from a C_17_H_10_O substructure through an inversion center at the centroid of the central quinone ring, generating a wide H-shaped mol­ecule, with a dihedral angle between the mean planes of the terminal benzene rings in each of the two symmetry-related pairs over the four mol­ecules of 68.6 (1) (*A*), 65.5 (4) (*B*), 62.3 (9) (*C*), and 65.8 (8)° (*D*), an average of 65.6 (1)°. This compound has applications in gas-separation membranes constructed from polymers of intrinsic microporosity (PIM). The title compound is a product of a double Diels–Alder reaction between anthracene and *p*-benzo­quinone followed by de­hydrogenation. It has also been characterized by cyclic voltammetry and rotating disc electrode polarography, FT–IR, high resolution mass spectrometry, elemental analysis, and ^1^H NMR.

## Abstract 2

5,7,12,14-Tetra­hydro-5,14:7,12-bis­([1,2]benzeno)­penta­cene-6,13-dione, used as a precursor in the synthesis of polymers of intrinsic microporosity (PIM) membranes, recrystallizes from DMF.

## Chemical context   

Pentiptycene and its derivatives are members of the iptycene family (Hart *et al.*, 1981 ▸). They possess a rigid, bulky, aromatic, three-dimensional scaffold which makes them suitable for specific applications in porous material construction (Yang & Swager, 1998*a* ▸), fluorescent polymers, chemical sensing (Yang & Swager, 1998*b* ▸) and mol­ecular machines (Sun *et al.*, 2010 ▸). The first iptycene derivative was reported 85 years ago (Clar, 1931 ▸). Pentiptycene, first prepared by Theilacker *et al.* (1960 ▸), is readily available from inexpensive materials and is made by Clar synthesis, which involves a Diels–Alder cyclo­addition between a polycyclic diene and a benzo­quinone followed by chloranil-induced de­hydrogenation. Pentiptycene quinone is a precursor for pentiptycene-6,13-diol, which is subsequently used as a principal reactant for polymer synthesis. Gong & Zhang (2011 ▸) synthesized poly(aryl­ene ether sulfone)s to fabricate highly conductive polymer electrolyte membranes for high-temperature and low-humidity conditions. Pentiptycene-based di­amines have been used in the preparation of polyimides with controlled mol­ecular cavities, for application in gas separation membranes (Luo *et al.*, 2015 ▸, 2016 ▸).

## Structural commentary   

**Figure 1:**
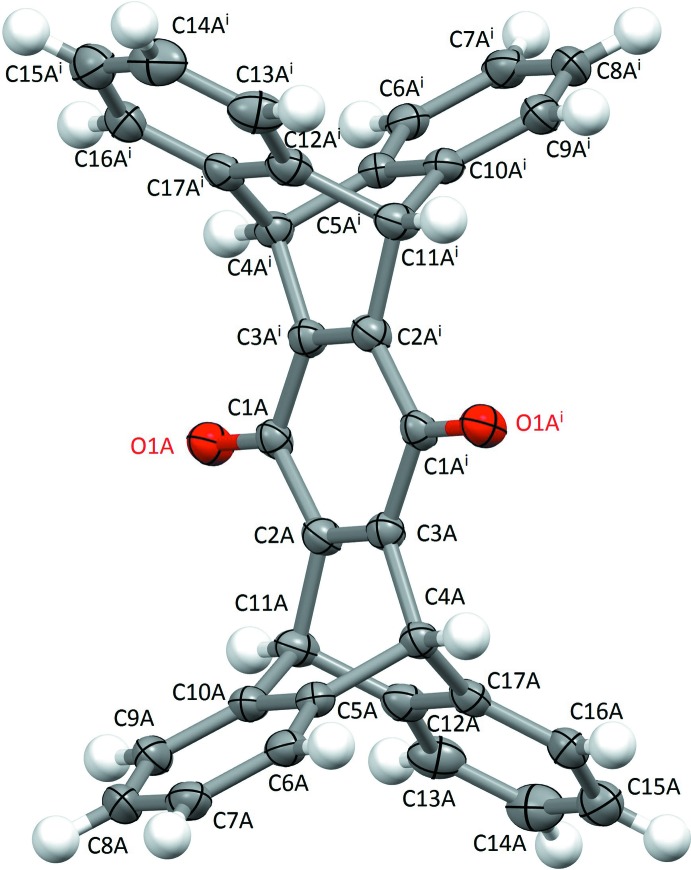
The structure of mol­ecule *A*, C_34_H_20_O_2_, one of four independent mol­ecules (*A*, *B*, *C*, and *D*) in the unit cell, showing the atom-labeling scheme with 30% probability ellipsoids. H atoms are rendered as spheres of arbitrary radius. An inversion center (1 − *x*, 1 − *y*, 1 − *z*) at the centroid of the central quinone ring generates the complete mol­ecule from a C_17_H_10_O substructure.

In the title compound, C_34_H_20_O_2_, four independent half-mol­ecules (*A*, *B*, *C*, *D*) crystallize in the asymmetric unit. An inversion center [1 − *x*, 1 − *y*, 2 − *z* (mol­ecule *A*), 1 − *x*, 2 − *y*, 1 − *z* (mol­ecule *B*), −*x*, 1 − *y*, 2 − *z* (mol­ecule *C*) and 2 − *x*, −*y*, 1 − *z* (mol­ecule *D*)] is present at the centroid of the central quinone ring in each mol­ecule and yields a C_17_H_10_O substructure, generating mol­ecules with a concave H-shape (Fig. 1 ▸).
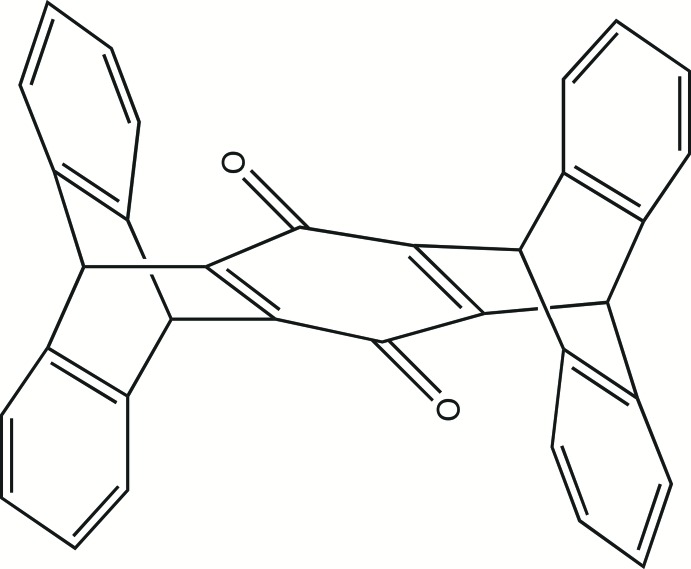


The dihedral angle between the mean planes of the terminal benzene rings in each of the symmetry-related sets over the four mol­ecules is (the complement of) 68.6 (1) (*A*), 65.5 (4) (*B*), 62.3 (9) (*C*) and 65.8 (8)° (*D*), an average of 65.6 (1)°. The three six-membered carbon rings fused between the benzene rings and the central quinone ring in each of the four mol­ecules adopt a boat conformation (Table 1 ▸). No classical hydrogen bonds are observed.

The central quinone moiety and H-shaped nature of the title compound make it very similar to its hydro­quinone analogue (Nozari *et al.*, 2016 ▸) which crystallized in a monoclinic (*P*2_1_/*n*) space group with a solvent DMF mol­ecule that generated O—H⋯O hydrogen bonds and weak C—H⋯O inter­molecular inter­actions in the crystal lattice. The average lengths of the C=O bonds in the title mol­ecule are shorter than the C—OH bond in the hydro­quinone, 1.219 (2) *vs* 1.3665 (16) Å, respectively. The average lengths of the C1—C2 and C2—C3 bonds in the central symmetry-generated quinone rings of the four mol­ecules are respectively 1.478 (1) and 1.344 (8) Å, while in the hydro­quinone analogue they are 1.395 (2) and 1.394 (2) Å. The average angle of the C1–C2–C3 group of the central core moiety of the four title quinone mol­ecules is 122.58 (16)°, whereas for the hydro­quinone analogue it is 117.31 (12)°. The oxidative conversion of the hydro­quinone to the quinone inevitably breaks the central ring’s aromaticity and localizes the remaining bonding π-electrons into the C=O and flanking (C2*A*—C3*A*) bonds. This phenomenon is typified by the comparison of a known hydro­quinone (also with hydrogen-bonded OH groups; Barnes *et al.* 1990 ▸) with a closely related quinone (Gautrot *et al.*, 2006 ▸). In the former case, the C—O single bonds are about 1.38 Å, while the ring C—C bonds are of like length. For the quinone, the C=O bonds are typically 1.22 Å, the four C—C bonds adjacent to C1*A* range from 1.48 to 1.50 Å, and the two C—C bonds flanking those in turn are 1.40 to 1.41 Å. In the hydro­quinone, the hydrogen bonds must nonetheless somewhat influence these bond lengths. In the quinone mol­ecule, only weak π–π ring inter­actions provide little if any influence toward the bonding motifs within the mol­ecule (Fig. 1 ▸).

## Supra­molecular features   

**Figure 2:**
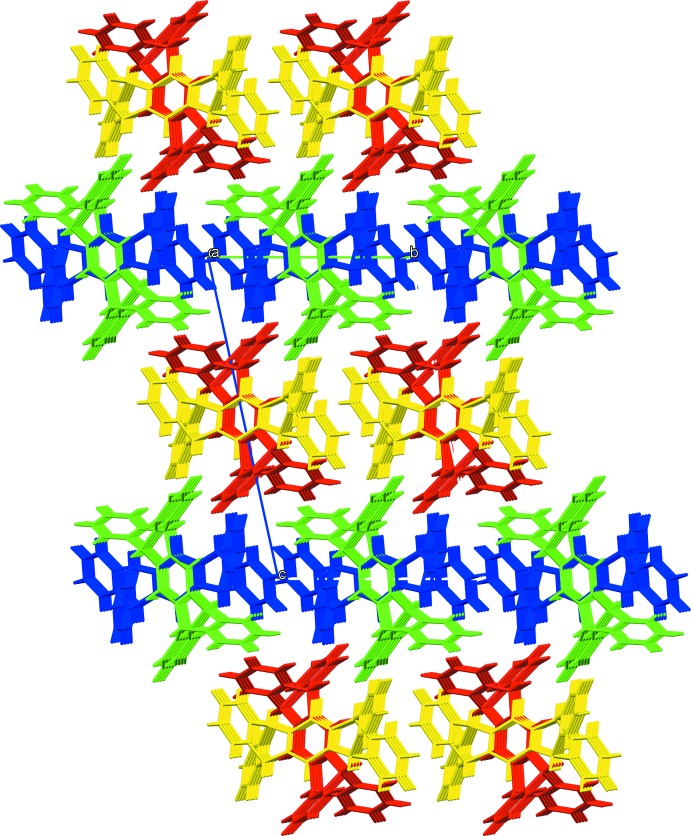
View of the crystal packing along the *a*-axis direction. The mol­ecules are color-coded as green (*A*), yellow (*B*), blue (*C*), and red (*D*). All four types of mol­ecules are arrayed along the *a*-axis direction, though none of the quinone planes is oriented simply parallel or perpendicular to the *a* axis. The *A* and *D* mol­ecules also form arrays along the *b*-axis direction more discernibly than other directions in the lattice.

In the crystal, there are four independent quinone mol­ecules oriented in different directions in the lattice. Despite the variation in orientation of the quinones with respect to one another, there are prominent arrays of the mol­ecules along the *a*-axis direction of the lattice (Figs. 2 ▸ and 3 ▸). The dihedral angles between the mean planes of the quinone rings, which emphasize the different orientations of the mol­ecules, range from 46 to 90°. While the hydrogen bonding found for the hydro­quinone is presumably a major lattice-structuring influence, we propose that the absence of such inter­actions for the quinone leads to a lattice geometry dominated by close packing of these exaggeratedly shaped quinone mol­ecules, and indeed the quinone crystal is more dense (1.338 g cm^−3^) than hydro­quinone (1.264 g cm^−3^). The crystal packing is influenced by weak π–π inter­molecular inter­actions involving the benzene rings from a flap of the V-shaped terminus of each of the mol­ecules *B* [C5*B*⋯C10*B*(1 − *x*, 1 − *y*, 1 − *z*) = 3.8375 (12) Å, ] and mol­ecules *C* [C5*C*⋯C10*C*(−*x*, 2 − *y*, 2 − *z*) = 3.9342 (12) Å]. Additional weak C—H⋯π inter­molecular inter­actions also contribute to the packing stability (Table 2 ▸).

## Electrochemistry   

**Figure 4:**
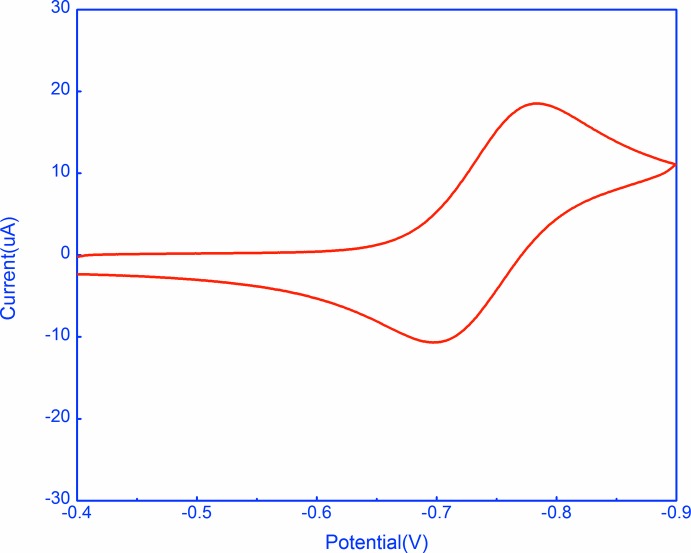
Cyclic voltammogram for reduction of 1 m*M* quinone *versus* the APE in DMF containing 0.1 *M* TBAPF_6_ as the supporting electrolyte, at a scan rate of 100 mV s^−1^. The APE potential is 340 mV more positive than that of the AgCl/Ag electrode (Pavlishchuk & Addison, 2000 ▸).

The quinone-hydro­quinone system is a prototype organic redox system; *Q* + e^−^ 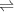 *Q* ^·−^, *Q* ^·−^ + e^−^ 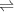 *Q* ^2−^. These systems have been studied electrochemically since the 1920s (Fieser, 1928 ▸). Cyclic voltammetry (CV) and rotating disc electrode (RDE) polarography were performed at 298 K on 1 m*M* quinone in DMF with 0.1 *M* tetra­butyl­ammonium hexa­fluorido­phosphate (TBAPF_6_) as the supporting electrolyte, at scan rates ranging from 50 to 10000 mV s^−1^ for CV, and 1200 to 3200 r.p.m. for the RDE. Experiments were run on a BASi–Epsilon instrument using a three-electrode cell incorporating a non-aqueous reference electrode (APE) (Pavlishchuk & Addison, 2000 ▸) and a 3 mm diameter Pt disc working electrode (Figs. 4 ▸ and 5 ▸). The first reduction to *Q* ^·−^ (*E*_1/2_^*a*^) was found by CV to *b* −0.741 (2) V, while formation of *Q* ^2−^ (*E*_1/2_^*b*^) was seen in the rotating disc polarogram at about −1.53 V; the RDE results also demonstrate unequivocally the reductive nature of these processes. The first reduction is reversible, with Δ*E*_p_° close to 59 mV, but the second reduction is complicated [similar outcomes have previously been observed for quinones in DMF solutions (Jeong *et al.*, 2000 ▸)]. The *E*_1/2_ values are within the range reported for quinone systems in the literature with *E*_1/2_^*a*^ ranging from −0.72 to −1.37 V and *E*_1/2_^*b*^ from −1.18 to −1.90 V *vs* AgCl/Ag (Bauscher & Mäntele 1992 ▸). From the CV results, the diffusion coefficient value of the title compound is estimated to be 5.4 × 10^−06^ cm^2^ s^−1^ in DMF, corresponding to a Dη value of 4.7 × 10 ^−08^ g cm s^−2^, consistent with the *n* = 1 assignment.

## Database survey   

X-ray structures for some hydro­quinone derivatives of the corresponding quinone compound have been reported. We recently described the undecorated hydro­quinone (Nozari *et al.*, 2016 ▸). Bis(tri­methyl­silylethyn­yl)pentiptycene was reported by Yang & Swager (1998*b* ▸), while a long-chain ether and an aryl­sulfonyl di­amide derivative were reported by Yang *et al.* (2000*a* ▸,*b* ▸). The hydro­quinone triflate ester was reported by Zyryanov *et al.* (2008 ▸), and a 4′-carb­oxy­benzyl ether derivative by Crane *et al.* (2013 ▸).

## Synthesis and crystallization   

**Figure 6:**
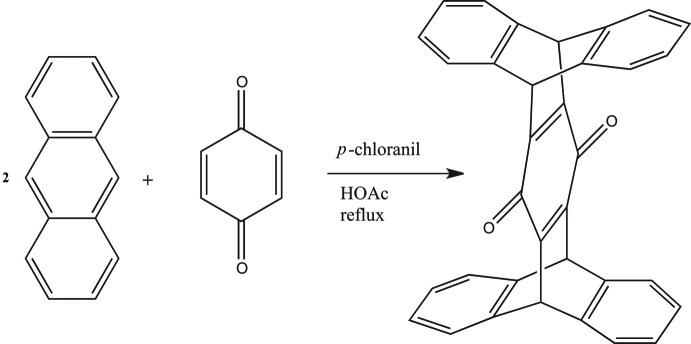
Synthesis of the title compound.

The title pentiptycene quinone was prepared using a double Diels–Alder reaction between anthracene and *p*-benzo­quinone (Fig. 6 ▸). The procedure reported by Cao *et al.* (2009 ▸) was followed. For this synthesis, 7.12 g (40 mmol) of anthracene and 2.16 g (20 mmol) of *p*-benzo­quinone were added to glacial acetic acid (250 mL), followed by addition of 9.84 g (40 mmol) of chloranil. The mixture was refluxed for 18 h, following which the solution was allowed to cool to room temperature. The resulting dark-yellow solid was filtered off, washed with diethyl ether, and vacuum desiccated, yielding the crude product (8.22 g, 89%), which was then recrystallized from DMF, washed with diethyl ether, and air-dried. Analysis calculated for C_34_H_20_O_2_: C, 88.7, H, 4.38. O, 6.95. Found: C, 88.4, H, 4.50, O, 7.09 (by difference). ^1^H NMR (500 MHz, chloro­form-*d*) δ 7.44–7.21 (*m*, 4H), 7.11–6.85 (*m*, 4H), 5.86 (*s*, 1H), 5.65 (*s*, 1H); FT–IR 1640 (C=O), 1579, 1456, 1293, 1200, 1137, 1019, 886, 742 cm^−1^; mass spectrum calculated for C_34_H_21_O_2_ (*m* + 1)^+^ *m*/*z* 461.154, found 461.153.

## Refinement   

Crystal data, data collection and structure refinement details are summarized in Table 3 ▸. All of the H atoms were refined using a riding-model approximation with C—H = 0.95 Å or 1.0 Å. Isotropic displacement parameters for these atoms were set to 1.2*U*_eq_ of the parent atom.

## Supplementary Material

Crystal structure: contains datablock(s) ta-mn1626, I. DOI: 10.1107/S2056989016017461/zl2675sup1.cif

Structure factors: contains datablock(s) I. DOI: 10.1107/S2056989016017461/zl2675Isup2.hkl

Supporting information file. DOI: 10.1107/S2056989016017461/zl2675Isup3.cml

CCDC reference: 1513684

Additional supporting information: crystallographic information; 3D view; checkCIF report

## Figures and Tables

**Figure 3:**
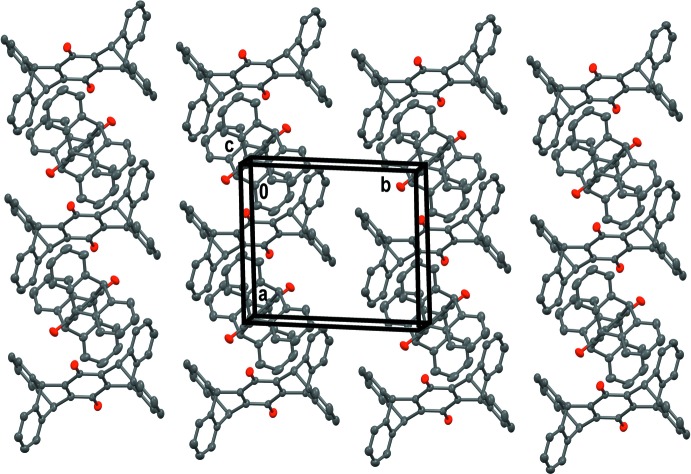
Crystal packing of the four independent mol­ecules (*A*, *B*, *C*, and *D*) viewed along along the *c* axis.

**Figure 5:**
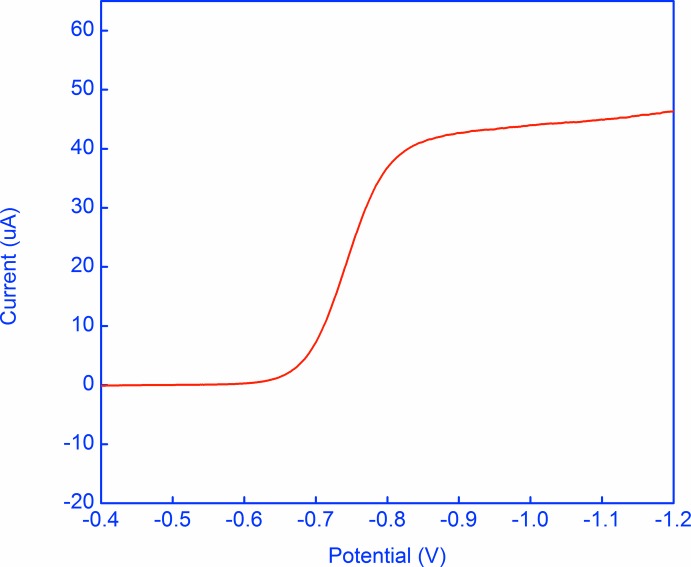
Rotating disc electrode polarogram for reduction of 1 m*M* quinone *versus* the APE in DMF containing 0.1 *M* TBAPF_6_ as the supporting electrolyte at a rotation rate of 2400 r.p.m. The APE potential is 340 mV more positive than the AgCl/Ag electrode (Pavlishchuk & Addison, 2000 ▸).

**Table 1:** Packing parameters (Å, °) for six-mol­ecule carbon rings in mol­ecules *A*, *B*, *C*, and *D*

Mol.	Carbon ring	*Q*	ω	φ
*A*	C2*A*–C5*A*/C10*A*/C11*A*	0.7952 (3)	89.74 (14)	300.20 (15)
*A*	C2*A*–C4*A*/C17*A*/C12*A*/C11*A*	0.788 (2)	89.94 (15)	119.27 (15)
*A*	C4*A*/C5*A*/C10*A*–C12*A*/C17*A*	0.845 (2)	89.67 (14)	359.59 (14)
*B*	C2*B*–C5*B*/C10*B*/C11*B*	0.809 (2)	89.94 (14)	120.14 (15)
*B*	C2*B*–C4*B*/C17*B*/C12*B*/C11*B*	0.790 (2)	89.82 (15)	300.15 (15)
*B*	C4*B*/C5*B*/C10*B*–C12*B*/C17*B*	0.845 (2)	89.67 (14)	359.59 (14)
*C*	C2*C*–C5*C*/C10*C*/C11*C*	0.798 (2)	89.77 (14)	119.61 (55)
*C*	C2*C*–C4*C*/C17*C*/C12*C*/C11*C*	0.805 (2)	89.91 (14)	300.43 (15)
*C*	C4*C*/C5*C*/C10*C*–C12*C*/C17*C*	0.818 (2)	90.17 (14)	180.42 (15)
*D*	C2*D*–C5*D*/C10*D*/C11*D*	0.789 (2)	90.00 (15)	119.35 (15)
*D*	C2*D*–C4*D*/C17*D*/C12*D*/C11*D*	0.800 (2)	89.42 (14)	300.04 (15)
*D*	C4*D*/C5*D*/C10*D*–C12*D*/C17*D*	0.833 (2)	90.53 (14)	179.83 (14)

**Table 2:** Weak C—H⋯π inter­molecular inter­actions (Å, °) *Cg*1, *Cg*2, *Cg*3 and *Cg*4 are the centroids of the C12*B*–C17*B*, C12*C*–C17*C*, C12*A*–C17*A* and C5*B*–C10*B* rings, respectively.

No.	*D*—H⋯*A*	*d*(*D*—H)	*d*(*D*⋯*A*)	<(*D*—H⋯*A*)
1	C8*B*—H8*B*⋯*Cg* ^i^	2.98	3.484 (2)	144
2	C7*C*—H7*C*⋯*Cg* ^ii^	2.70	3.417 (2)	133
3	C16*C*—H16*C*⋯*Cg* ^i^	2.74	3.662 (3)	165
4	C4*D*—H4*D*⋯*Cg* ^ii^	2.98	3.948 (2)	163

Symmetry codes: (i) 1 − *x*, 1 − *y*, 1 − *z*; (ii) −*x*, 2 − *y*, 2 − *z*.

**Table 3:** Experimental details

Crystal data	
Chemical formula	C_34_H_20_O_2_
*M*_r_	460.50
Crystal system, space group	Triclinic, *P* 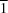
Temperature (K)	173
*a*, *b*, *c* (Å)	10.3419 (4), 11.7885 (6), 19.2267 (11)
α, β, γ (°)	77.606 (5), 89.306 (4), 86.658 (4)
*V* (Å^3^)	2285.5 (2)
*Z*	4
Radiation type	Cu *K*α
μ (mm^−1^)	0.64
Crystal size (mm)	0.38 × 0.14 × 0.08

Data collection	
Diffractometer	Rigaku Oxford Diffaction Eos Gemini
Absorption correction	Multi-scan (*CrysAlis PRO* and *CrysAlis RED*; Rigaku OD, 2012 ▸)
No. of measured, independent and observed [*I* > 2σ(*I*)] reflections	16688, 8703, 7068
*R*_int_	0.038
(sin θ/λ)_max_ (Å^−1^)	0.615

Refinement	
*R*[*F* ^2^ > 2σ(*F* ^2^)], *wR*(*F* ^2^), *S*	0.057, 0.161, 1.05
No. of reflections	8703
No. of parameters	649
H-atom treatment	H-atom parameters constrained
Δρ_max_, Δρ_min_ (e Å^−3^)	0.41, −0.31

Computer programs: *CrysAlis PRO* and *CrysAlis RED* (Rigaku OD, 2012 ▸), *SHELXT* (Sheldrick, 2015*a* ▸), *SHELXL* (Sheldrick, 2015*b* ▸) and *OLEX2* (Dolomanov *et al.*, 2009 ▸).

## supplementary crystallographic information

### Crystal data

**Table:**

C_34_H_20_O_2_	*Z* = 4
*M**_r_* = 460.50	*F*(000) = 960
Triclinic, *P*1	*D*_x_ = 1.338 Mg m^−^^3^
*a* = 10.3419 (4) Å	Cu *K*α radiation, λ = 1.54184 Å
*b* = 11.7885 (6) Å	Cell parameters from 2655 reflections
*c* = 19.2267 (11) Å	θ = 3.9–71.4°
α = 77.606 (5)°	µ = 0.64 mm^−^^1^
β = 89.306 (4)°	*T* = 173 K
γ = 86.658 (4)°	Prism, yellow
*V* = 2285.5 (2) Å^3^	0.38 × 0.14 × 0.08 mm

### Data collection

**Table:**

Rigaku Oxford Diffaction Eos Gemini diffractometer	8703 independent reflections
Radiation source: Enhance (Cu) X-ray Source	7068 reflections with *I* > 2σ(*I*)
Graphite monochromator	*R*_int_ = 0.038
Detector resolution: 16.0416 pixels mm^-1^	θ_max_ = 71.5°, θ_min_ = 3.9°
ω scans	*h* = −12→6
Absorption correction: multi-scan (CrysAlis PRO and CrysAlis RED; Rigaku OD, 2012)	*k* = −14→14
	*l* = −23→21
16688 measured reflections	

### Refinement

**Table:**

Refinement on *F*^2^	Primary atom site location: structure-invariant direct methods
Least-squares matrix: full	Hydrogen site location: inferred from neighbouring sites
*R*[*F*^2^ > 2σ(*F*^2^)] = 0.057	H-atom parameters constrained
*wR*(*F*^2^) = 0.161	*w* = 1/[σ^2^(*F*_o_^2^) + (0.0938*P*)^2^ + 0.3137*P*] where *P* = (*F*_o_^2^ + 2*F*_c_^2^)/3
*S* = 1.05	(Δ/σ)_max_ = 0.001
8703 reflections	Δρ_max_ = 0.41 e Å^−^^3^
649 parameters	Δρ_min_ = −0.31 e Å^−^^3^
0 restraints	

### Special details

**Table:**

Experimental. ^1^H NMR (500 MHz, chloroform-*d*) δ 7.44-7.21 (m, 4H), 7.11-6.85 (m, 4H), 5.86 (s,1H), 5.65 (s, 1H). ; IR 1640 (C=O), 1579, 1456, 1293, 1200, 1137, 1019, 886, 742 cm^-1^; mass spectrum calcd for C_34_H_21_O_2_ (m+1)^+^ m/z 461.1536, found 461.1529 (4).
Geometry. All esds (except the esd in the dihedral angle between two l.s. planes) are estimated using the full covariance matrix. The cell esds are taken into account individually in the estimation of esds in distances, angles and torsion angles; correlations between esds in cell parameters are only used when they are defined by crystal symmetry. An approximate (isotropic) treatment of cell esds is used for estimating esds involving l.s. planes.

### Fractional atomic coordinates and isotropic or equivalent isotropic displacement parameters (Å^2^)

**Table:**

	*x*	*y*	*z*	*U*_iso_*/*U*_eq_	
O1A	0.67598 (14)	0.64743 (13)	0.93841 (7)	0.0337 (3)	
C1A	0.59748 (17)	0.57908 (15)	0.96675 (9)	0.0229 (3)	
C2A	0.52423 (17)	0.51065 (16)	0.92620 (9)	0.0235 (4)	
C3A	0.43357 (18)	0.43896 (16)	0.95639 (9)	0.0249 (4)	
C4A	0.36776 (19)	0.37687 (17)	0.90550 (10)	0.0282 (4)	
H4A	0.2998	0.3244	0.9287	0.034*	
C5A	0.31656 (19)	0.47330 (18)	0.84369 (10)	0.0300 (4)	
C6A	0.1929 (2)	0.4858 (2)	0.81612 (12)	0.0374 (5)	
H6A	0.1283	0.4352	0.8372	0.045*	
C7A	0.1644 (2)	0.5749 (2)	0.75612 (13)	0.0448 (6)	
H7A	0.0795	0.5851	0.7366	0.054*	
C8A	0.2583 (2)	0.6477 (2)	0.72533 (12)	0.0423 (5)	
H8A	0.2380	0.7070	0.6844	0.051*	
C9A	0.3831 (2)	0.63521 (19)	0.75371 (10)	0.0345 (4)	
H9A	0.4475	0.6864	0.7330	0.041*	
C10A	0.41176 (19)	0.54718 (17)	0.81254 (10)	0.0282 (4)	
C11A	0.54511 (18)	0.51449 (16)	0.84738 (9)	0.0262 (4)	
H11A	0.6135	0.5676	0.8255	0.031*	
C12A	0.57305 (19)	0.38856 (17)	0.84075 (9)	0.0271 (4)	
C13A	0.6777 (2)	0.3465 (2)	0.80621 (10)	0.0333 (4)	
H13A	0.7430	0.3965	0.7854	0.040*	
C14A	0.6855 (2)	0.2293 (2)	0.80242 (12)	0.0426 (5)	
H14A	0.7570	0.1993	0.7791	0.051*	
C15A	0.5903 (3)	0.1565 (2)	0.83218 (12)	0.0442 (5)	
H15A	0.5964	0.0771	0.8288	0.053*	
C16A	0.4848 (2)	0.19892 (19)	0.86725 (11)	0.0366 (5)	
H16A	0.4195	0.1489	0.8879	0.044*	
C17A	0.47715 (19)	0.31436 (17)	0.87140 (10)	0.0285 (4)	
O1C	−0.10862 (14)	0.53082 (12)	0.87054 (7)	0.0301 (3)	
C1C	−0.06219 (17)	0.51755 (15)	0.93001 (9)	0.0233 (3)	
C2C	−0.00715 (18)	0.61326 (16)	0.95641 (9)	0.0249 (4)	
C3C	0.05255 (18)	0.59679 (16)	1.01992 (10)	0.0258 (4)	
C4C	0.10523 (19)	0.70623 (16)	1.03611 (10)	0.0289 (4)	
H4C	0.1498	0.6928	1.0833	0.035*	
C5C	−0.01092 (19)	0.79431 (16)	1.02907 (10)	0.0285 (4)	
C6C	−0.0546 (2)	0.85554 (18)	1.07934 (11)	0.0365 (5)	
H6C	−0.0122	0.8448	1.1240	0.044*	
C7C	−0.1621 (2)	0.93336 (19)	1.06320 (14)	0.0427 (5)	
H7C	−0.1930	0.9762	1.0972	0.051*	
C8C	−0.2243 (2)	0.94899 (19)	0.99840 (14)	0.0419 (5)	
H8C	−0.2979	1.0019	0.9884	0.050*	
C9C	−0.1802 (2)	0.88804 (17)	0.94772 (12)	0.0343 (4)	
H9C	−0.2225	0.8994	0.9030	0.041*	
C10C	−0.07360 (19)	0.81053 (16)	0.96344 (10)	0.0275 (4)	
C11C	−0.01140 (19)	0.73785 (16)	0.91421 (10)	0.0267 (4)	
H11C	−0.0565	0.7490	0.8673	0.032*	
C12C	0.12981 (19)	0.76996 (16)	0.90756 (10)	0.0285 (4)	
C13C	0.1940 (2)	0.81458 (17)	0.84526 (12)	0.0352 (4)	
H13C	0.1508	0.8272	0.8007	0.042*	
C14C	0.3226 (2)	0.8409 (2)	0.84845 (14)	0.0434 (5)	
H14C	0.3674	0.8715	0.8057	0.052*	
C15C	0.3864 (2)	0.82302 (19)	0.91309 (15)	0.0432 (5)	
H15C	0.4745	0.8411	0.9146	0.052*	
C16C	0.3209 (2)	0.77832 (17)	0.97621 (13)	0.0357 (5)	
H16C	0.3642	0.7658	1.0208	0.043*	
C17C	0.1929 (2)	0.75262 (16)	0.97313 (11)	0.0294 (4)	
O1D	1.16824 (14)	−0.17465 (13)	0.55887 (7)	0.0339 (3)	
C1D	1.09380 (17)	−0.09392 (16)	0.53194 (9)	0.0237 (4)	
C2D	1.01696 (18)	−0.02375 (16)	0.57483 (9)	0.0247 (4)	
C3D	0.92860 (18)	0.06052 (16)	0.54596 (9)	0.0249 (4)	
C4D	0.85470 (18)	0.11861 (17)	0.59908 (9)	0.0271 (4)	
H4D	0.7887	0.1806	0.5767	0.033*	
C5D	0.95849 (19)	0.16186 (17)	0.64165 (9)	0.0282 (4)	
C6D	0.9632 (2)	0.27375 (18)	0.65312 (11)	0.0365 (5)	
H6D	0.8994	0.3325	0.6331	0.044*	
C7D	1.0633 (3)	0.2985 (2)	0.69466 (13)	0.0451 (5)	
H7D	1.0679	0.3748	0.7028	0.054*	
C8D	1.1557 (2)	0.2126 (2)	0.72407 (12)	0.0418 (5)	
H8D	1.2233	0.2305	0.7523	0.050*	
C9D	1.1507 (2)	0.09976 (19)	0.71257 (10)	0.0337 (4)	
H9D	1.2143	0.0409	0.7328	0.040*	
C10D	1.05177 (19)	0.07511 (17)	0.67134 (9)	0.0282 (4)	
C11D	1.02761 (19)	−0.04370 (16)	0.65518 (9)	0.0274 (4)	
H11D	1.0943	−0.1062	0.6761	0.033*	
C12D	0.8906 (2)	−0.06857 (17)	0.68249 (9)	0.0287 (4)	
C13D	0.8553 (2)	−0.16378 (18)	0.73347 (10)	0.0352 (5)	
H13D	0.9179	−0.2240	0.7530	0.042*	
C14D	0.7271 (3)	−0.1700 (2)	0.75565 (12)	0.0451 (6)	
H14D	0.7021	−0.2340	0.7913	0.054*	
C15D	0.6355 (2)	−0.0831 (2)	0.72593 (13)	0.0472 (6)	
H15D	0.5479	−0.0888	0.7411	0.057*	
C16D	0.6699 (2)	0.0127 (2)	0.67405 (12)	0.0378 (5)	
H16D	0.6068	0.0721	0.6538	0.045*	
C17D	0.79803 (19)	0.01873 (18)	0.65302 (10)	0.0293 (4)	
O1B	0.36832 (14)	0.94275 (12)	0.62240 (7)	0.0332 (3)	
C1B	0.42572 (18)	0.96821 (16)	0.56576 (10)	0.0248 (4)	
C2B	0.50197 (18)	0.88122 (16)	0.53524 (10)	0.0258 (4)	
C3B	0.57432 (19)	0.91049 (16)	0.47596 (10)	0.0278 (4)	
C4B	0.6456 (2)	0.80840 (16)	0.45309 (11)	0.0303 (4)	
H4B	0.6989	0.8316	0.4091	0.036*	
C5B	0.5396 (2)	0.72624 (16)	0.44506 (10)	0.0295 (4)	
C6B	0.5156 (2)	0.68273 (18)	0.38563 (11)	0.0371 (5)	
H6B	0.5659	0.7041	0.3437	0.044*	
C7B	0.4165 (3)	0.60711 (18)	0.38805 (12)	0.0414 (5)	
H7B	0.3988	0.5771	0.3473	0.050*	
C8B	0.3432 (2)	0.57501 (17)	0.44943 (12)	0.0366 (5)	
H8B	0.2767	0.5224	0.4506	0.044*	
C9B	0.36681 (19)	0.61968 (16)	0.50941 (11)	0.0304 (4)	
H9B	0.3165	0.5984	0.5514	0.036*	
C10B	0.46500 (19)	0.69565 (15)	0.50657 (10)	0.0263 (4)	
C11B	0.50641 (18)	0.75110 (15)	0.56702 (10)	0.0259 (4)	
H11B	0.4524	0.7302	0.6109	0.031*	
C12B	0.64921 (19)	0.71316 (16)	0.57920 (10)	0.0281 (4)	
C13B	0.7044 (2)	0.64890 (17)	0.64158 (11)	0.0324 (4)	
H13B	0.6533	0.6281	0.6832	0.039*	
C14B	0.8359 (2)	0.61507 (19)	0.64261 (13)	0.0406 (5)	
H14B	0.8745	0.5708	0.6852	0.049*	
C15B	0.9105 (2)	0.6454 (2)	0.58232 (15)	0.0443 (5)	
H15B	1.0002	0.6225	0.5837	0.053*	
C16B	0.8548 (2)	0.70972 (19)	0.51912 (13)	0.0395 (5)	
H16B	0.9061	0.7300	0.4774	0.047*	
C17B	0.7247 (2)	0.74353 (16)	0.51782 (11)	0.0314 (4)	

### Atomic displacement parameters (Å^2^)

**Table:**

	*U*^11^	*U*^22^	*U*^33^	*U*^12^	*U*^13^	*U*^23^
O1A	0.0373 (7)	0.0377 (7)	0.0279 (7)	−0.0125 (6)	0.0087 (6)	−0.0090 (6)
C1A	0.0230 (8)	0.0239 (8)	0.0218 (8)	0.0010 (6)	0.0017 (6)	−0.0055 (6)
C2A	0.0258 (8)	0.0257 (8)	0.0185 (8)	0.0022 (7)	−0.0004 (6)	−0.0043 (6)
C3A	0.0290 (9)	0.0254 (8)	0.0205 (8)	0.0003 (7)	−0.0015 (7)	−0.0062 (7)
C4A	0.0315 (9)	0.0322 (9)	0.0229 (9)	−0.0051 (8)	0.0003 (7)	−0.0093 (7)
C5A	0.0317 (10)	0.0370 (10)	0.0242 (9)	0.0027 (8)	−0.0016 (7)	−0.0143 (8)
C6A	0.0311 (10)	0.0506 (13)	0.0360 (11)	0.0023 (9)	−0.0031 (8)	−0.0228 (10)
C7A	0.0403 (12)	0.0579 (14)	0.0411 (12)	0.0187 (10)	−0.0179 (10)	−0.0264 (11)
C8A	0.0538 (14)	0.0442 (12)	0.0289 (10)	0.0175 (10)	−0.0114 (9)	−0.0128 (9)
C9A	0.0442 (11)	0.0366 (10)	0.0224 (9)	0.0101 (9)	−0.0022 (8)	−0.0088 (8)
C10A	0.0336 (10)	0.0324 (10)	0.0193 (8)	0.0049 (8)	−0.0014 (7)	−0.0088 (7)
C11A	0.0294 (9)	0.0299 (9)	0.0189 (8)	−0.0008 (7)	0.0016 (7)	−0.0046 (7)
C12A	0.0327 (9)	0.0318 (9)	0.0169 (8)	0.0029 (7)	−0.0040 (7)	−0.0069 (7)
C13A	0.0329 (10)	0.0452 (12)	0.0225 (9)	0.0037 (8)	−0.0007 (7)	−0.0102 (8)
C14A	0.0487 (13)	0.0496 (13)	0.0321 (11)	0.0122 (10)	−0.0008 (9)	−0.0185 (10)
C15A	0.0659 (15)	0.0347 (11)	0.0353 (11)	0.0069 (10)	−0.0038 (10)	−0.0168 (9)
C16A	0.0479 (12)	0.0337 (10)	0.0305 (10)	−0.0041 (9)	−0.0022 (8)	−0.0116 (8)
C17A	0.0336 (10)	0.0323 (10)	0.0207 (8)	0.0006 (8)	−0.0037 (7)	−0.0090 (7)
O1C	0.0384 (7)	0.0295 (7)	0.0221 (6)	−0.0014 (5)	−0.0059 (5)	−0.0046 (5)
C1C	0.0258 (8)	0.0239 (8)	0.0202 (8)	0.0015 (7)	0.0004 (6)	−0.0057 (7)
C2C	0.0304 (9)	0.0226 (8)	0.0211 (8)	0.0008 (7)	0.0011 (7)	−0.0039 (7)
C3C	0.0322 (9)	0.0228 (8)	0.0228 (8)	−0.0003 (7)	−0.0020 (7)	−0.0062 (7)
C4C	0.0389 (10)	0.0232 (9)	0.0249 (9)	−0.0021 (8)	−0.0058 (7)	−0.0052 (7)
C5C	0.0367 (10)	0.0214 (8)	0.0279 (9)	−0.0047 (7)	0.0011 (7)	−0.0058 (7)
C6C	0.0506 (12)	0.0300 (10)	0.0321 (10)	−0.0096 (9)	0.0057 (9)	−0.0118 (8)
C7C	0.0489 (13)	0.0310 (10)	0.0536 (14)	−0.0067 (9)	0.0149 (10)	−0.0208 (10)
C8C	0.0378 (11)	0.0284 (10)	0.0613 (15)	0.0013 (8)	0.0041 (10)	−0.0147 (10)
C9C	0.0359 (10)	0.0248 (9)	0.0415 (11)	−0.0013 (8)	−0.0020 (8)	−0.0056 (8)
C10C	0.0326 (10)	0.0216 (8)	0.0288 (9)	−0.0044 (7)	0.0016 (7)	−0.0056 (7)
C11C	0.0360 (10)	0.0217 (8)	0.0220 (8)	−0.0002 (7)	−0.0023 (7)	−0.0038 (7)
C12C	0.0360 (10)	0.0208 (8)	0.0290 (9)	0.0010 (7)	0.0015 (7)	−0.0071 (7)
C13C	0.0459 (12)	0.0270 (9)	0.0337 (10)	−0.0010 (8)	0.0088 (9)	−0.0092 (8)
C14C	0.0447 (12)	0.0342 (11)	0.0516 (14)	−0.0014 (9)	0.0189 (10)	−0.0113 (10)
C15C	0.0289 (10)	0.0311 (10)	0.0700 (16)	0.0010 (8)	0.0060 (10)	−0.0128 (10)
C16C	0.0348 (10)	0.0231 (9)	0.0497 (12)	0.0012 (8)	−0.0076 (9)	−0.0090 (8)
C17C	0.0357 (10)	0.0208 (8)	0.0320 (10)	0.0019 (7)	−0.0029 (8)	−0.0067 (7)
O1D	0.0385 (8)	0.0355 (7)	0.0259 (7)	0.0091 (6)	−0.0053 (5)	−0.0055 (6)
C1D	0.0273 (9)	0.0243 (8)	0.0193 (8)	−0.0026 (7)	−0.0007 (6)	−0.0040 (7)
C2D	0.0310 (9)	0.0253 (8)	0.0174 (8)	−0.0034 (7)	0.0012 (6)	−0.0034 (7)
C3D	0.0293 (9)	0.0253 (8)	0.0204 (8)	−0.0019 (7)	0.0009 (6)	−0.0053 (7)
C4D	0.0317 (9)	0.0300 (9)	0.0201 (8)	0.0011 (7)	0.0028 (7)	−0.0072 (7)
C5D	0.0369 (10)	0.0307 (9)	0.0178 (8)	−0.0046 (8)	0.0053 (7)	−0.0066 (7)
C6D	0.0520 (13)	0.0292 (10)	0.0293 (10)	−0.0018 (9)	0.0042 (9)	−0.0092 (8)
C7D	0.0640 (15)	0.0377 (11)	0.0388 (12)	−0.0126 (11)	0.0021 (10)	−0.0174 (9)
C8D	0.0492 (13)	0.0498 (13)	0.0310 (10)	−0.0160 (10)	−0.0001 (9)	−0.0151 (9)
C9D	0.0377 (11)	0.0412 (11)	0.0224 (9)	−0.0071 (9)	0.0018 (7)	−0.0060 (8)
C10D	0.0368 (10)	0.0312 (9)	0.0170 (8)	−0.0060 (8)	0.0052 (7)	−0.0050 (7)
C11D	0.0357 (10)	0.0274 (9)	0.0187 (8)	−0.0006 (7)	−0.0009 (7)	−0.0045 (7)
C12D	0.0402 (11)	0.0309 (9)	0.0168 (8)	−0.0075 (8)	0.0021 (7)	−0.0080 (7)
C13D	0.0551 (13)	0.0323 (10)	0.0208 (9)	−0.0156 (9)	0.0011 (8)	−0.0078 (8)
C14D	0.0660 (15)	0.0462 (13)	0.0281 (10)	−0.0286 (12)	0.0119 (10)	−0.0127 (9)
C15D	0.0449 (13)	0.0669 (16)	0.0379 (12)	−0.0250 (12)	0.0169 (10)	−0.0238 (11)
C16D	0.0360 (11)	0.0499 (13)	0.0326 (10)	−0.0089 (9)	0.0061 (8)	−0.0184 (9)
C17D	0.0351 (10)	0.0347 (10)	0.0210 (8)	−0.0059 (8)	0.0038 (7)	−0.0115 (7)
O1B	0.0418 (8)	0.0287 (7)	0.0293 (7)	−0.0056 (6)	0.0113 (6)	−0.0063 (5)
C1B	0.0289 (9)	0.0236 (8)	0.0233 (8)	−0.0068 (7)	0.0023 (7)	−0.0071 (7)
C2B	0.0311 (9)	0.0227 (8)	0.0240 (9)	−0.0044 (7)	0.0010 (7)	−0.0053 (7)
C3B	0.0342 (10)	0.0245 (9)	0.0260 (9)	−0.0040 (7)	0.0046 (7)	−0.0081 (7)
C4B	0.0397 (10)	0.0226 (9)	0.0295 (9)	−0.0038 (8)	0.0099 (8)	−0.0074 (7)
C5B	0.0416 (11)	0.0201 (8)	0.0267 (9)	0.0002 (7)	0.0002 (8)	−0.0055 (7)
C6B	0.0603 (14)	0.0259 (9)	0.0251 (9)	0.0007 (9)	0.0003 (9)	−0.0066 (7)
C7B	0.0686 (15)	0.0263 (10)	0.0310 (11)	0.0003 (10)	−0.0140 (10)	−0.0102 (8)
C8B	0.0432 (12)	0.0235 (9)	0.0434 (12)	−0.0026 (8)	−0.0130 (9)	−0.0070 (8)
C9B	0.0348 (10)	0.0212 (8)	0.0342 (10)	0.0008 (7)	−0.0037 (8)	−0.0040 (7)
C10B	0.0332 (9)	0.0200 (8)	0.0255 (9)	0.0012 (7)	−0.0023 (7)	−0.0047 (7)
C11B	0.0320 (9)	0.0218 (8)	0.0239 (9)	−0.0040 (7)	0.0018 (7)	−0.0045 (7)
C12B	0.0326 (10)	0.0224 (8)	0.0318 (10)	−0.0051 (7)	0.0000 (7)	−0.0107 (7)
C13B	0.0390 (11)	0.0286 (9)	0.0320 (10)	−0.0041 (8)	−0.0050 (8)	−0.0106 (8)
C14B	0.0429 (12)	0.0336 (11)	0.0468 (13)	−0.0035 (9)	−0.0122 (10)	−0.0114 (9)
C15B	0.0302 (10)	0.0376 (11)	0.0674 (16)	−0.0019 (9)	−0.0061 (10)	−0.0160 (11)
C16B	0.0373 (11)	0.0298 (10)	0.0528 (13)	−0.0072 (8)	0.0094 (9)	−0.0114 (9)
C17B	0.0365 (10)	0.0232 (9)	0.0359 (10)	−0.0054 (8)	0.0036 (8)	−0.0084 (8)

### Geometric parameters (Å, º)

**Table:**

O1A—C1A	1.217 (2)	O1D—C1D	1.216 (2)
C1A—C2A	1.479 (3)	C1D—C2D	1.482 (3)
C1A—C3A^i^	1.481 (2)	C1D—C3D^iii^	1.482 (2)
C2A—C3A	1.341 (3)	C2D—C3D	1.342 (3)
C2A—C11A	1.520 (2)	C2D—C11D	1.516 (2)
C3A—C1A^i^	1.481 (2)	C3D—C1D^iii^	1.482 (2)
C3A—C4A	1.529 (2)	C3D—C4D	1.522 (2)
C4A—H4A	1.0000	C4D—H4D	1.0000
C4A—C5A	1.533 (3)	C4D—C5D	1.532 (3)
C4A—C17A	1.531 (3)	C4D—C17D	1.532 (3)
C5A—C6A	1.379 (3)	C5D—C6D	1.387 (3)
C5A—C10A	1.396 (3)	C5D—C10D	1.396 (3)
C6A—H6A	0.9500	C6D—H6D	0.9500
C6A—C7A	1.405 (3)	C6D—C7D	1.396 (3)
C7A—H7A	0.9500	C7D—H7D	0.9500
C7A—C8A	1.377 (4)	C7D—C8D	1.383 (4)
C8A—H8A	0.9500	C8D—H8D	0.9500
C8A—C9A	1.395 (3)	C8D—C9D	1.399 (3)
C9A—H9A	0.9500	C9D—H9D	0.9500
C9A—C10A	1.383 (3)	C9D—C10D	1.383 (3)
C10A—C11A	1.533 (3)	C10D—C11D	1.533 (3)
C11A—H11A	1.0000	C11D—H11D	1.0000
C11A—C12A	1.528 (3)	C11D—C12D	1.527 (3)
C12A—C13A	1.386 (3)	C12D—C13D	1.386 (3)
C12A—C17A	1.401 (3)	C12D—C17D	1.395 (3)
C13A—H13A	0.9500	C13D—H13D	0.9500
C13A—C14A	1.397 (3)	C13D—C14D	1.389 (3)
C14A—H14A	0.9500	C14D—H14D	0.9500
C14A—C15A	1.381 (4)	C14D—C15D	1.386 (4)
C15A—H15A	0.9500	C15D—H15D	0.9500
C15A—C16A	1.399 (3)	C15D—C16D	1.396 (4)
C16A—H16A	0.9500	C16D—H16D	0.9500
C16A—C17A	1.378 (3)	C16D—C17D	1.383 (3)
O1C—C1C	1.221 (2)	O1B—C1B	1.222 (2)
C1C—C2C	1.477 (3)	C1B—C2B	1.473 (3)
C1C—C3C^ii^	1.477 (2)	C1B—C3B^iv^	1.482 (3)
C2C—C3C	1.346 (3)	C2B—C3B	1.349 (3)
C2C—C11C	1.516 (2)	C2B—C11B	1.523 (2)
C3C—C1C^ii^	1.478 (2)	C3B—C1B^iv^	1.482 (3)
C3C—C4C	1.522 (3)	C3B—C4B	1.517 (3)
C4C—H4C	1.0000	C4B—H4B	1.0000
C4C—C5C	1.529 (3)	C4B—C5B	1.535 (3)
C4C—C17C	1.528 (3)	C4B—C17B	1.532 (3)
C5C—C6C	1.382 (3)	C5B—C6B	1.380 (3)
C5C—C10C	1.397 (3)	C5B—C10B	1.397 (3)
C6C—H6C	0.9500	C6B—H6B	0.9500
C6C—C7C	1.393 (3)	C6B—C7B	1.390 (3)
C7C—H7C	0.9500	C7B—H7B	0.9500
C7C—C8C	1.381 (4)	C7B—C8B	1.390 (3)
C8C—H8C	0.9500	C8B—H8B	0.9500
C8C—C9C	1.388 (3)	C8B—C9B	1.396 (3)
C9C—H9C	0.9500	C9B—H9B	0.9500
C9C—C10C	1.383 (3)	C9B—C10B	1.386 (3)
C10C—C11C	1.521 (3)	C10B—C11B	1.527 (3)
C11C—H11C	1.0000	C11B—H11B	1.0000
C11C—C12C	1.527 (3)	C11B—C12B	1.524 (3)
C12C—C13C	1.380 (3)	C12B—C13B	1.384 (3)
C12C—C17C	1.397 (3)	C12B—C17B	1.400 (3)
C13C—H13C	0.9500	C13B—H13B	0.9500
C13C—C14C	1.388 (3)	C13B—C14B	1.394 (3)
C14C—H14C	0.9500	C14B—H14B	0.9500
C14C—C15C	1.384 (4)	C14B—C15B	1.378 (4)
C15C—H15C	0.9500	C15B—H15B	0.9500
C15C—C16C	1.398 (3)	C15B—C16B	1.398 (4)
C16C—H16C	0.9500	C16B—H16B	0.9500
C16C—C17C	1.380 (3)	C16B—C17B	1.380 (3)
			
O1A—C1A—C2A	122.10 (16)	O1D—C1D—C2D	122.37 (16)
O1A—C1A—C3A^i^	122.37 (17)	O1D—C1D—C3D^iii^	122.32 (17)
C2A—C1A—C3A^i^	115.52 (16)	C2D—C1D—C3D^iii^	115.30 (16)
C1A—C2A—C11A	123.36 (16)	C1D—C2D—C11D	123.09 (16)
C3A—C2A—C1A	122.48 (16)	C3D—C2D—C1D	122.69 (16)
C3A—C2A—C11A	114.16 (16)	C3D—C2D—C11D	114.19 (17)
C1A^i^—C3A—C4A	123.36 (16)	C1D^iii^—C3D—C4D	123.13 (16)
C2A—C3A—C1A^i^	121.99 (17)	C2D—C3D—C1D^iii^	121.93 (17)
C2A—C3A—C4A	114.64 (16)	C2D—C3D—C4D	114.94 (16)
C3A—C4A—H4A	113.6	C3D—C4D—H4D	113.8
C3A—C4A—C5A	105.66 (15)	C3D—C4D—C5D	105.49 (15)
C3A—C4A—C17A	105.90 (15)	C3D—C4D—C17D	105.10 (15)
C5A—C4A—H4A	113.6	C5D—C4D—H4D	113.8
C17A—C4A—H4A	113.6	C5D—C4D—C17D	103.74 (15)
C17A—C4A—C5A	103.40 (15)	C17D—C4D—H4D	113.8
C6A—C5A—C4A	125.9 (2)	C6D—C5D—C4D	125.99 (19)
C6A—C5A—C10A	120.8 (2)	C6D—C5D—C10D	120.70 (19)
C10A—C5A—C4A	113.12 (17)	C10D—C5D—C4D	113.29 (17)
C5A—C6A—H6A	120.7	C5D—C6D—H6D	120.6
C5A—C6A—C7A	118.6 (2)	C5D—C6D—C7D	118.9 (2)
C7A—C6A—H6A	120.7	C7D—C6D—H6D	120.6
C6A—C7A—H7A	119.7	C6D—C7D—H7D	119.8
C8A—C7A—C6A	120.6 (2)	C8D—C7D—C6D	120.5 (2)
C8A—C7A—H7A	119.7	C8D—C7D—H7D	119.8
C7A—C8A—H8A	119.7	C7D—C8D—H8D	119.7
C7A—C8A—C9A	120.6 (2)	C7D—C8D—C9D	120.6 (2)
C9A—C8A—H8A	119.7	C9D—C8D—H8D	119.7
C8A—C9A—H9A	120.5	C8D—C9D—H9D	120.5
C10A—C9A—C8A	119.0 (2)	C10D—C9D—C8D	119.0 (2)
C10A—C9A—H9A	120.5	C10D—C9D—H9D	120.5
C5A—C10A—C11A	113.17 (17)	C5D—C10D—C11D	113.14 (17)
C9A—C10A—C5A	120.45 (19)	C9D—C10D—C5D	120.35 (19)
C9A—C10A—C11A	126.28 (19)	C9D—C10D—C11D	126.48 (19)
C2A—C11A—C10A	105.63 (15)	C2D—C11D—C10D	105.91 (14)
C2A—C11A—H11A	113.5	C2D—C11D—H11D	113.6
C2A—C11A—C12A	106.17 (14)	C2D—C11D—C12D	104.82 (15)
C10A—C11A—H11A	113.5	C10D—C11D—H11D	113.6
C12A—C11A—C10A	103.76 (15)	C12D—C11D—C10D	104.51 (15)
C12A—C11A—H11A	113.5	C12D—C11D—H11D	113.6
C13A—C12A—C11A	126.30 (19)	C13D—C12D—C11D	126.31 (19)
C13A—C12A—C17A	120.31 (19)	C13D—C12D—C17D	120.29 (19)
C17A—C12A—C11A	113.34 (17)	C17D—C12D—C11D	113.35 (16)
C12A—C13A—H13A	120.6	C12D—C13D—H13D	120.4
C12A—C13A—C14A	118.9 (2)	C12D—C13D—C14D	119.2 (2)
C14A—C13A—H13A	120.6	C14D—C13D—H13D	120.4
C13A—C14A—H14A	119.6	C13D—C14D—H14D	119.9
C15A—C14A—C13A	120.7 (2)	C15D—C14D—C13D	120.2 (2)
C15A—C14A—H14A	119.6	C15D—C14D—H14D	119.9
C14A—C15A—H15A	119.8	C14D—C15D—H15D	119.4
C14A—C15A—C16A	120.4 (2)	C14D—C15D—C16D	121.1 (2)
C16A—C15A—H15A	119.8	C16D—C15D—H15D	119.4
C15A—C16A—H16A	120.5	C15D—C16D—H16D	120.9
C17A—C16A—C15A	119.1 (2)	C17D—C16D—C15D	118.2 (2)
C17A—C16A—H16A	120.5	C17D—C16D—H16D	120.9
C12A—C17A—C4A	112.86 (17)	C12D—C17D—C4D	113.24 (17)
C16A—C17A—C4A	126.48 (19)	C16D—C17D—C4D	125.70 (19)
C16A—C17A—C12A	120.63 (19)	C16D—C17D—C12D	121.03 (19)
O1C—C1C—C2C	122.70 (17)	O1B—C1B—C2B	122.41 (17)
O1C—C1C—C3C^ii^	122.33 (17)	O1B—C1B—C3B^iv^	122.03 (17)
C2C—C1C—C3C^ii^	114.93 (15)	C2B—C1B—C3B^iv^	115.54 (16)
C1C—C2C—C11C	123.12 (16)	C1B—C2B—C11B	123.61 (16)
C3C—C2C—C1C	122.65 (17)	C3B—C2B—C1B	122.50 (17)
C3C—C2C—C11C	114.23 (16)	C3B—C2B—C11B	113.90 (17)
C1C^ii^—C3C—C4C	123.37 (16)	C1B^iv^—C3B—C4B	123.54 (16)
C2C—C3C—C1C^ii^	122.35 (17)	C2B—C3B—C1B^iv^	121.81 (17)
C2C—C3C—C4C	114.26 (16)	C2B—C3B—C4B	114.48 (17)
C3C—C4C—H4C	113.5	C3B—C4B—H4B	113.5
C3C—C4C—C5C	105.24 (16)	C3B—C4B—C5B	104.83 (16)
C3C—C4C—C17C	105.07 (15)	C3B—C4B—C17B	106.07 (16)
C5C—C4C—H4C	113.5	C5B—C4B—H4B	113.5
C17C—C4C—H4C	113.5	C17B—C4B—H4B	113.5
C17C—C4C—C5C	105.15 (15)	C17B—C4B—C5B	104.71 (15)
C6C—C5C—C4C	126.77 (19)	C6B—C5B—C4B	126.60 (19)
C6C—C5C—C10C	120.41 (19)	C6B—C5B—C10B	120.60 (19)
C10C—C5C—C4C	112.82 (17)	C10B—C5B—C4B	112.80 (17)
C5C—C6C—H6C	120.6	C5B—C6B—H6B	120.5
C5C—C6C—C7C	118.7 (2)	C5B—C6B—C7B	119.0 (2)
C7C—C6C—H6C	120.6	C7B—C6B—H6B	120.5
C6C—C7C—H7C	119.6	C6B—C7B—H7B	119.7
C8C—C7C—C6C	120.8 (2)	C8B—C7B—C6B	120.68 (19)
C8C—C7C—H7C	119.6	C8B—C7B—H7B	119.7
C7C—C8C—H8C	119.7	C7B—C8B—H8B	119.8
C7C—C8C—C9C	120.6 (2)	C7B—C8B—C9B	120.38 (19)
C9C—C8C—H8C	119.7	C9B—C8B—H8B	119.8
C8C—C9C—H9C	120.6	C8B—C9B—H9B	120.6
C10C—C9C—C8C	118.9 (2)	C10B—C9B—C8B	118.7 (2)
C10C—C9C—H9C	120.6	C10B—C9B—H9B	120.6
C5C—C10C—C11C	113.41 (17)	C5B—C10B—C11B	113.23 (16)
C9C—C10C—C5C	120.59 (19)	C9B—C10B—C5B	120.62 (18)
C9C—C10C—C11C	125.99 (18)	C9B—C10B—C11B	126.13 (18)
C2C—C11C—C10C	105.50 (15)	C2B—C11B—C10B	105.02 (14)
C2C—C11C—H11C	113.5	C2B—C11B—H11B	113.4
C2C—C11C—C12C	105.00 (15)	C2B—C11B—C12B	105.99 (15)
C10C—C11C—H11C	113.5	C10B—C11B—H11B	113.4
C10C—C11C—C12C	105.13 (15)	C12B—C11B—C10B	104.96 (15)
C12C—C11C—H11C	113.5	C12B—C11B—H11B	113.4
C13C—C12C—C11C	126.32 (18)	C13B—C12B—C11B	126.45 (18)
C13C—C12C—C17C	120.44 (19)	C13B—C12B—C17B	120.27 (19)
C17C—C12C—C11C	113.22 (17)	C17B—C12B—C11B	113.16 (17)
C12C—C13C—H13C	120.4	C12B—C13B—H13B	120.4
C12C—C13C—C14C	119.2 (2)	C12B—C13B—C14B	119.3 (2)
C14C—C13C—H13C	120.4	C14B—C13B—H13B	120.4
C13C—C14C—H14C	119.6	C13B—C14B—H14B	119.7
C15C—C14C—C13C	120.8 (2)	C15B—C14B—C13B	120.5 (2)
C15C—C14C—H14C	119.6	C15B—C14B—H14B	119.7
C14C—C15C—H15C	120.0	C14B—C15B—H15B	119.8
C14C—C15C—C16C	119.9 (2)	C14B—C15B—C16B	120.4 (2)
C16C—C15C—H15C	120.0	C16B—C15B—H15B	119.8
C15C—C16C—H16C	120.3	C15B—C16B—H16B	120.3
C17C—C16C—C15C	119.3 (2)	C17B—C16B—C15B	119.4 (2)
C17C—C16C—H16C	120.3	C17B—C16B—H16B	120.3
C12C—C17C—C4C	112.92 (17)	C12B—C17B—C4B	112.86 (17)
C16C—C17C—C4C	126.77 (18)	C16B—C17B—C4B	126.84 (19)
C16C—C17C—C12C	120.30 (19)	C16B—C17B—C12B	120.2 (2)
			
O1A—C1A—C2A—C3A	−177.04 (18)	O1D—C1D—C2D—C3D	−175.59 (18)
O1A—C1A—C2A—C11A	3.3 (3)	O1D—C1D—C2D—C11D	2.1 (3)
C1A—C2A—C3A—C1A^i^	−1.6 (3)	C1D—C2D—C3D—C1D^iii^	−3.3 (3)
C1A—C2A—C3A—C4A	179.42 (15)	C1D—C2D—C3D—C4D	176.98 (16)
C1A—C2A—C11A—C10A	−124.72 (18)	C1D—C2D—C11D—C10D	127.96 (18)
C1A—C2A—C11A—C12A	125.51 (18)	C1D—C2D—C11D—C12D	−121.86 (18)
C1A^i^—C3A—C4A—C5A	126.87 (18)	C1D^iii^—C3D—C4D—C5D	−124.63 (18)
C1A^i^—C3A—C4A—C17A	−123.85 (18)	C1D^iii^—C3D—C4D—C17D	126.10 (18)
C2A—C3A—C4A—C5A	−54.1 (2)	C2D—C3D—C4D—C5D	55.1 (2)
C2A—C3A—C4A—C17A	55.1 (2)	C2D—C3D—C4D—C17D	−54.2 (2)
C2A—C11A—C12A—C13A	−127.88 (19)	C2D—C11D—C12D—C13D	127.22 (19)
C2A—C11A—C12A—C17A	54.7 (2)	C2D—C11D—C12D—C17D	−55.4 (2)
C3A^i^—C1A—C2A—C3A	1.5 (3)	C3D^iii^—C1D—C2D—C3D	3.1 (3)
C3A^i^—C1A—C2A—C11A	−178.16 (16)	C3D^iii^—C1D—C2D—C11D	−179.23 (16)
C3A—C2A—C11A—C10A	55.6 (2)	C3D—C2D—C11D—C10D	−54.2 (2)
C3A—C2A—C11A—C12A	−54.1 (2)	C3D—C2D—C11D—C12D	56.0 (2)
C3A—C4A—C5A—C6A	−130.6 (2)	C3D—C4D—C5D—C6D	128.4 (2)
C3A—C4A—C5A—C10A	53.7 (2)	C3D—C4D—C5D—C10D	−53.14 (19)
C3A—C4A—C17A—C12A	−53.0 (2)	C3D—C4D—C17D—C12D	53.2 (2)
C3A—C4A—C17A—C16A	129.0 (2)	C3D—C4D—C17D—C16D	−128.7 (2)
C4A—C5A—C6A—C7A	−175.77 (19)	C4D—C5D—C6D—C7D	178.60 (19)
C4A—C5A—C10A—C9A	176.68 (17)	C4D—C5D—C10D—C9D	−178.70 (16)
C4A—C5A—C10A—C11A	0.1 (2)	C4D—C5D—C10D—C11D	−0.7 (2)
C5A—C4A—C17A—C12A	57.9 (2)	C5D—C4D—C17D—C12D	−57.3 (2)
C5A—C4A—C17A—C16A	−120.1 (2)	C5D—C4D—C17D—C16D	120.8 (2)
C5A—C6A—C7A—C8A	0.4 (3)	C5D—C6D—C7D—C8D	−0.3 (3)
C5A—C10A—C11A—C2A	−54.5 (2)	C5D—C10D—C11D—C2D	54.5 (2)
C5A—C10A—C11A—C12A	56.94 (19)	C5D—C10D—C11D—C12D	−55.92 (19)
C6A—C5A—C10A—C9A	0.7 (3)	C6D—C5D—C10D—C9D	−0.2 (3)
C6A—C5A—C10A—C11A	−175.96 (17)	C6D—C5D—C10D—C11D	177.79 (17)
C6A—C7A—C8A—C9A	−0.8 (3)	C6D—C7D—C8D—C9D	0.1 (4)
C7A—C8A—C9A—C10A	1.1 (3)	C7D—C8D—C9D—C10D	0.0 (3)
C8A—C9A—C10A—C5A	−1.1 (3)	C8D—C9D—C10D—C5D	0.0 (3)
C8A—C9A—C10A—C11A	175.08 (18)	C8D—C9D—C10D—C11D	−177.64 (18)
C9A—C10A—C11A—C2A	129.1 (2)	C9D—C10D—C11D—C2D	−127.7 (2)
C9A—C10A—C11A—C12A	−119.4 (2)	C9D—C10D—C11D—C12D	121.9 (2)
C10A—C5A—C6A—C7A	−0.3 (3)	C10D—C5D—C6D—C7D	0.3 (3)
C10A—C11A—C12A—C13A	121.0 (2)	C10D—C11D—C12D—C13D	−121.6 (2)
C10A—C11A—C12A—C17A	−56.41 (19)	C10D—C11D—C12D—C17D	55.80 (19)
C11A—C2A—C3A—C1A^i^	178.10 (16)	C11D—C2D—C3D—C1D^iii^	178.84 (16)
C11A—C2A—C3A—C4A	−0.9 (2)	C11D—C2D—C3D—C4D	−0.9 (2)
C11A—C12A—C13A—C14A	−177.13 (18)	C11D—C12D—C13D—C14D	176.09 (18)
C11A—C12A—C17A—C4A	−1.0 (2)	C11D—C12D—C17D—C4D	0.9 (2)
C11A—C12A—C17A—C16A	177.15 (17)	C11D—C12D—C17D—C16D	−177.26 (17)
C12A—C13A—C14A—C15A	0.4 (3)	C12D—C13D—C14D—C15D	1.4 (3)
C13A—C12A—C17A—C4A	−178.57 (16)	C13D—C12D—C17D—C4D	178.51 (17)
C13A—C12A—C17A—C16A	−0.5 (3)	C13D—C12D—C17D—C16D	0.3 (3)
C13A—C14A—C15A—C16A	−0.6 (3)	C13D—C14D—C15D—C16D	−0.8 (3)
C14A—C15A—C16A—C17A	0.3 (3)	C14D—C15D—C16D—C17D	−0.1 (3)
C15A—C16A—C17A—C4A	178.09 (19)	C15D—C16D—C17D—C4D	−177.67 (19)
C15A—C16A—C17A—C12A	0.3 (3)	C15D—C16D—C17D—C12D	0.3 (3)
C17A—C4A—C5A—C6A	118.4 (2)	C17D—C4D—C5D—C6D	−121.3 (2)
C17A—C4A—C5A—C10A	−57.4 (2)	C17D—C4D—C5D—C10D	57.1 (2)
C17A—C12A—C13A—C14A	0.2 (3)	C17D—C12D—C13D—C14D	−1.1 (3)
O1C—C1C—C2C—C3C	−174.98 (18)	O1B—C1B—C2B—C3B	−174.18 (18)
O1C—C1C—C2C—C11C	4.3 (3)	O1B—C1B—C2B—C11B	6.0 (3)
C1C—C2C—C3C—C1C^ii^	−3.1 (3)	C1B—C2B—C3B—C1B^iv^	−4.7 (3)
C1C—C2C—C3C—C4C	178.55 (17)	C1B—C2B—C3B—C4B	179.98 (17)
C1C—C2C—C11C—C10C	125.93 (18)	C1B—C2B—C11B—C10B	124.21 (19)
C1C—C2C—C11C—C12C	−123.29 (18)	C1B—C2B—C11B—C12B	−125.00 (18)
C1C^ii^—C3C—C4C—C5C	−122.69 (19)	C1B^iv^—C3B—C4B—C5B	−119.42 (19)
C1C^ii^—C3C—C4C—C17C	126.58 (18)	C1B^iv^—C3B—C4B—C17B	130.11 (18)
C2C—C3C—C4C—C5C	55.7 (2)	C2B—C3B—C4B—C5B	55.8 (2)
C2C—C3C—C4C—C17C	−55.1 (2)	C2B—C3B—C4B—C17B	−54.6 (2)
C2C—C11C—C12C—C13C	126.79 (19)	C2B—C11B—C12B—C13B	129.40 (19)
C2C—C11C—C12C—C17C	−54.7 (2)	C2B—C11B—C12B—C17B	−54.5 (2)
C3C^ii^—C1C—C2C—C3C	2.9 (3)	C3B^iv^—C1B—C2B—C3B	4.4 (3)
C3C^ii^—C1C—C2C—C11C	−177.90 (16)	C3B^iv^—C1B—C2B—C11B	−175.40 (16)
C3C—C2C—C11C—C10C	−54.8 (2)	C3B—C2B—C11B—C10B	−55.6 (2)
C3C—C2C—C11C—C12C	56.0 (2)	C3B—C2B—C11B—C12B	55.2 (2)
C3C—C4C—C5C—C6C	126.4 (2)	C3B—C4B—C5B—C6B	125.7 (2)
C3C—C4C—C5C—C10C	−54.2 (2)	C3B—C4B—C5B—C10B	−55.0 (2)
C3C—C4C—C17C—C12C	54.8 (2)	C3B—C4B—C17B—C12B	53.9 (2)
C3C—C4C—C17C—C16C	−126.0 (2)	C3B—C4B—C17B—C16B	−129.6 (2)
C4C—C5C—C6C—C7C	179.44 (19)	C4B—C5B—C6B—C7B	178.78 (19)
C4C—C5C—C10C—C9C	−179.44 (17)	C4B—C5B—C10B—C9B	−178.40 (17)
C4C—C5C—C10C—C11C	−0.5 (2)	C4B—C5B—C10B—C11B	0.0 (2)
C5C—C4C—C17C—C12C	−55.9 (2)	C5B—C4B—C17B—C12B	−56.7 (2)
C5C—C4C—C17C—C16C	123.2 (2)	C5B—C4B—C17B—C16B	119.9 (2)
C5C—C6C—C7C—C8C	0.2 (3)	C5B—C6B—C7B—C8B	−0.4 (3)
C5C—C10C—C11C—C2C	54.9 (2)	C5B—C10B—C11B—C2B	55.0 (2)
C5C—C10C—C11C—C12C	−55.8 (2)	C5B—C10B—C11B—C12B	−56.57 (19)
C6C—C5C—C10C—C9C	0.0 (3)	C6B—C5B—C10B—C9B	1.0 (3)
C6C—C5C—C10C—C11C	179.03 (17)	C6B—C5B—C10B—C11B	179.43 (18)
C6C—C7C—C8C—C9C	−0.5 (3)	C6B—C7B—C8B—C9B	0.9 (3)
C7C—C8C—C9C—C10C	0.6 (3)	C7B—C8B—C9B—C10B	−0.5 (3)
C8C—C9C—C10C—C5C	−0.4 (3)	C8B—C9B—C10B—C5B	−0.5 (3)
C8C—C9C—C10C—C11C	−179.21 (19)	C8B—C9B—C10B—C11B	−178.72 (17)
C9C—C10C—C11C—C2C	−126.2 (2)	C9B—C10B—C11B—C2B	−126.70 (19)
C9C—C10C—C11C—C12C	123.1 (2)	C9B—C10B—C11B—C12B	121.8 (2)
C10C—C5C—C6C—C7C	0.0 (3)	C10B—C5B—C6B—C7B	−0.5 (3)
C10C—C11C—C12C—C13C	−122.2 (2)	C10B—C11B—C12B—C13B	−119.8 (2)
C10C—C11C—C12C—C17C	56.3 (2)	C10B—C11B—C12B—C17B	56.32 (19)
C11C—C2C—C3C—C1C^ii^	177.63 (16)	C11B—C2B—C3B—C1B^iv^	175.14 (16)
C11C—C2C—C3C—C4C	−0.8 (2)	C11B—C2B—C3B—C4B	−0.2 (2)
C11C—C12C—C13C—C14C	179.07 (18)	C11B—C12B—C13B—C14B	176.00 (18)
C11C—C12C—C17C—C4C	−0.4 (2)	C11B—C12B—C17B—C4B	0.3 (2)
C11C—C12C—C17C—C16C	−179.57 (17)	C11B—C12B—C17B—C16B	−176.46 (18)
C12C—C13C—C14C—C15C	−0.1 (3)	C12B—C13B—C14B—C15B	0.2 (3)
C13C—C12C—C17C—C4C	178.23 (17)	C13B—C12B—C17B—C4B	176.70 (17)
C13C—C12C—C17C—C16C	−1.0 (3)	C13B—C12B—C17B—C16B	−0.1 (3)
C13C—C14C—C15C—C16C	−0.2 (3)	C13B—C14B—C15B—C16B	−0.5 (3)
C14C—C15C—C16C—C17C	−0.1 (3)	C14B—C15B—C16B—C17B	0.6 (3)
C15C—C16C—C17C—C4C	−178.44 (18)	C15B—C16B—C17B—C4B	−176.58 (19)
C15C—C16C—C17C—C12C	0.7 (3)	C15B—C16B—C17B—C12B	−0.3 (3)
C17C—C4C—C5C—C6C	−123.0 (2)	C17B—C4B—C5B—C6B	−122.9 (2)
C17C—C4C—C5C—C10C	56.5 (2)	C17B—C4B—C5B—C10B	56.4 (2)
C17C—C12C—C13C—C14C	0.7 (3)	C17B—C12B—C13B—C14B	0.2 (3)

Symmetry codes: (i) −*x*+1, −*y*+1, −*z*+2; (ii) −*x*, −*y*+1, −*z*+2; (iii) −*x*+2, −*y*, −*z*+1; (iv) −*x*+1, −*y*+2, −*z*+1.

## References

[bb1] Barnes, J. C., Paton, J. D. & Blyth, C. S. (1990). *Acta Cryst.* C**46**, 1183–1184.

[bb2] Bauscher, M. & Mäntele, W. (1992). *J. Phys. Chem.* **96**, 11101–11108.

[bb3] Cao, J., Lu, H. Y. & Chen, C. F. (2009). *Tetrahedron*, **65**, 8104–8112.

[bb4] Clar, E. (1931). *Ber. Dtsch. Chem. Ges. A/B*, **64**, 1676–1688.

[bb5] Crane, A. K., Wong, E. Y. L. & MacLachlan, M. J. (2013). *CrystEngComm*, **15**, 9811–9819.

[bb6] Dolomanov, O. V., Bourhis, L. J., Gildea, R. J., Howard, J. A. K. & Puschmann, H. (2009). *J. Appl. Cryst.* **42**, 339–341.

[bb7] Fieser, L. P. (1928). *J. Am. Chem. Soc.* **50**, 439–465.

[bb8] Gautrot, J. E., Hodge, O., Cupertino, D. & Helliwell, M. (2006). *New J. Chem.* **30**, 1801–1807.

[bb9] Gong, F. & Zhang, S. (2011). *J. Power Sources*, **196**, 9876–9883.

[bb10] Hart, H., Shamouilian, S. & Takehira, Y. (1981). *J. Org. Chem.* **46**, 4427–4432.

[bb11] Jeong, H., Choi, E. M., Kang, S. O., Nam, K. C. & Jeon, S. (2000). *J. Electroanal. Chem.* **485**, 154–160.

[bb12] Luo, S., Liu, Q., Zhang, B., Wiegand, J. R., Freeman, B. D. & Guo, R. (2015). *J. Membr. Sci.* **480**, 20–30.

[bb13] Luo, S., Wiegand, J. R., Kazanowska, B., Doherty, C. M., Konstas, K., Hill, A. J. & Guo, R. (2016). *Macromolecules*, **49**, 3395–3405.

[bb14] Nozari, M., Kaur, M., Jasinski, J. P., Addison, A. W., Arabi Shamsabadi, A. & Soroush, M. (2016). *IUCrData*, **1**, x161130. 10.1107/S2056989016017461 PMC5137597 27980819

[bb15] Pavlishchuk, V. V. & Addison, A. W. (2000). *Inorg. Chim. Acta*, **298**, 97–102.

[bb16] Rigaku OD (2012). *CrysAlis PRO* and *CrysAlis RED*. Rigaku Americas Corporation, The Woodlands, Texas, USA.

[bb17] Sheldrick, G. M. (2015*a*). *Acta Cryst.* A**71**, 3–8.

[bb18] Sheldrick, G. M. (2015*b*). *Acta Cryst.* C**71**, 3–8.

[bb19] Sun, W. T., Huang, Y. T., Huang, G. J., Lu, H. F., Chao, I., Huang, S. L., Huang, S. J., Lin, Y. C., Ho, J. H. & Yang, J. S. (2010). *Chem. Eur. J.* **16**, 11594–11604. 10.1002/chem.201000764 20827691

[bb20] Theilacker, W., Berger-Brose, U. & Beyer, K. H. (1960). *Chem. Ber.* **93**, 1658–1681.

[bb21] Yang, J. S., Lee, C. C., Yau, S. L., Chang, C. C., Lee, C. C. & Leu, J. M. (2000*a*). *J. Org. Chem.* **65**, 871–877.

[bb22] Yang, J. S., Liu, C. & Lee, G. (2000*b*). *Tetrahedron Lett.* **41**, 7911–7915.

[bb23] Yang, J. S. & Swager, T. M. (1998*a*). *J. Am. Chem. Soc.* **120**, 11864–11873.

[bb24] Yang, J. S. & Swager, T. M. (1998*b*). *J. Am. Chem. Soc.* **120**, 5321–5322.

[bb25] Zyryanov, G. V., Palacios, M. A. & Anzenbacher, P. Jr (2008). *Org. Lett.* **10**, 3681–3684. 10.1021/ol801030u 18656945

